# Inducement of positive nutritional practices through health promotion campaigns among parents/caregivers in Albania

**DOI:** 10.1093/eurpub/ckad182

**Published:** 2023-10-24

**Authors:** Suela Vasil, Iris Mone, Albano Alia, Kliton Muça, Eni Tresa, Genc Burazeri

**Affiliations:** “Schools for Health”, A Project of the Swiss Development and Cooperation (SDC) Agency, Tirana, Albania; Department of Informatics, Faculty of Natural Sciences, University of Tirana, Tirana, Albania; Faculty of Medicine, University of Medicine, Tirana, Albania; “Schools for Health”, A Project of the Swiss Development and Cooperation (SDC) Agency, Tirana, Albania; “Schools for Health”, A Project of the Swiss Development and Cooperation (SDC) Agency, Tirana, Albania; “Schools for Health”, A Project of the Swiss Development and Cooperation (SDC) Agency, Tirana, Albania; “Schools for Health”, A Project of the Swiss Development and Cooperation (SDC) Agency, Tirana, Albania; Faculty of Medicine, University of Medicine, Tirana, Albania; Department of International Health, CAPHRI (Care and Public Health Research Institute), Maastricht University, Maastricht, The Netherlands

## Abstract

Opting for homemade meals is the healthiest choice. We assessed the change in nutritional practices among parents/caregivers exposed to health promotion campaigns. Pre- and post-intervention surveys inquiring about nutritional practices were conducted respectively in March and June 2022 in a community-based sample of 583 parents/caregivers in Albania (62% females; age: 39.7 ± 7.1 years; response: 83%). The multi-component intervention consisted of community-based ‘onsite’ events (awareness raising campaigns) and ‘online’ interventions (knowledge portal and digital applications). After the intervention, the prevalence of home cooking and/or provision of home-made foods to children for eating at school increased by 11% (both *P* < 0.01). Engagement in healthy nutritional practices ‘only after the intervention’ increased especially among Roma/Egyptian parents/caregivers.

## Introduction

Different forms of malnutrition persist among children globally,^1^ with an increasing trend of overweight and obesity,^1^^,^^2^ which is currently recognized as the main consequence of unhealthy nutritional habits worldwide.^1–3^

Regardless of the specific recipe, home-cooked food tends to be healthier than restaurant food, prepared grocery store meals, or different food items available in kiosks and/or vending machines.^4^ Home preparation may also encourage/motivate children to take home-made foods at school.^5^ This is very important especially for countries where no meals are provided in school settings.

A considerable proportion of Albanian parents/caregivers lack adequate knowledge about healthy nutritional practices.^6^^,^^7^

Traditionally, home cooking (including all daily meals) has been common in most Albanian families, a habit which has been gradually decreasing in the past few decades, especially in large urban areas.^6^^,^^7^ This has led to an increase in ultra-processed food consumption, especially in urban areas.^6^^,^^7^ Of note, neither free nor paid meals are currently offered in public schools in Albania. Thus, bringing food from home is essential for ensuring healthy nutritional habits among schoolchildren in Albania. Otherwise, children purchase energy-dense, nutrient-poor food at the nearby school kiosks.^6^

‘Schools for Health’ (S4H) is a project of the Swiss Development and Cooperation (SDC) Agency, implemented by Save the Children in Albania during the period 2021–5 (http://shkollatpershendetin.al/en/). The aim of the project is to induce positive behavioural changes in the Albanian population, with a particular focus on schoolchildren aged 6–15 years and their respective parents/caregivers. The project works ‘onsite’ in selected schools and communities of Albania through a wide array of interventions targeting children and their parents/caregivers. In addition, the project operates at a national level, implementing multi-component ‘online’ interventions by use of a knowledge portal and digital applications aiming at promoting a wide range of healthy behaviours.

In this context, our aim was to assess the overall impact of S4H’s interventions aiming at promoting, among other things, healthy nutritional practices in Albanian parents/caregivers.

## Methods

The study population consisted of a representative sample of parents/caregivers from three regions of Albania included in onsite interventions by S4H in 2022: Lezha (north), Durrës (central) and Berat (south Albania).

A pre- and post-intervention study was conducted in the same sample of parents/caregivers [before the ‘intervention’ in March 2022 and next in June 2022, after a 4-month exposure to project’s interventions (onsite activities and online interventions) aimed at promoting healthy nutritional practices].

The multi-component ‘intervention’ consisted of periodic informative sessions with parents/caregivers regarding healthy nutritional practices, positive parenting sessions, and community-based awareness raising campaigns about the importance of healthy nutrition. Furthermore, parents/caregivers were exposed to other project interventions involving their respective children (food fairs, cooking classes and other activities conducted at school and/or community level, and online interventions using a knowledge portal and two digital applications specifically developed for promoting healthy nutritional practices).

In both survey rounds (i.e. before and after the ‘intervention’), an anonymous and structured self-administered questionnaire inquired parents/caregivers about the following specific nutritional habits: (i) whether they usually cooked at home; (ii) whether they usually provided home-made foods to their children for eating at school. Potential answers for each question were: ‘yes’ vs. ‘no’. In addition, socio-demographic data were collected.

The study was approved by the Albanian Ministry of Education and Sport in February 2022. All participants were informed about the aim and procedures of the study and were explained in sufficient detail particularly the aspects related to anonymousness of the surveys and the successive aggregated data analysis.

Two-related samples McNemar test was used to assess the change in nutritional practices after the intervention, whereas binary logistic regression was employed to assess the association of socio-demographic characteristics with changes in nutritional practices ‘only after the intervention’.

## Results

The study sample (*n* = 583; response rate: 83%) consisted of ∼62% females and had an overall mean age of 39.7 ± 7.1 years.

The prevalence of home cooking and provision of home-made foods to children for eating at school increased respectively from 80% and 82% before the intervention to 91% and 93% after the intervention (both *P* < 0.01).

Cooking at home ‘only after the intervention’ was evidenced in 106 (18%) of the overall number of study participants (table 1, upper panel). It was significantly higher among men compared to women [odds ratio (OR) = 2.2, 95% confidence interval (CI) = 1.5–3.4]. Furthermore, cooking at home only after the intervention was significantly higher among participants belonging to Roma/Egyptian minorities compared with ethnic Albanians (OR = 2.1, 95% CI = 1.1–4.0). Conversely, there was no relationship with age, or urban/rural residence.

**Table 1:** Association of engagement in healthy nutritional practices ‘only after the intervention’ with socio-demographic factors of the parents/caregivers

Upper panel: cooking at home ‘only after the intervention’					
Variable	No (*N* = 477)	Yes (*N* = 106)	**OR** ^a^	**95% CI** ^a^	***P*** ^a^
**Age (years)**	39.5 ± 7.3^b^	40.4 ± 6.5	1.02	0.99–1.05	0.276
**Gender**	164 (34.4)^c^	57 (53.8)			<0.001
Men	313 (65.6)	49 (46.2)	2.22	1.45–3.40	
Women			1.00	Reference	
**Place of residence**					0.486
Rural areas	163 (34.2)	40 (37.7)	1.17	0.76–1.81	
Urban areas	314 (65.8)	66 (62.3)	1.00	Reference	
**Ethnicity**	35 (7.3)	15 (14.2)			0.026
Roma/Egyptian	442 (92.7)	91 (85.8)	2.08	1.09–3.97	
Ethnic Albanian			1.00	Reference	

Lower panel: giving food to their children for eating at school ‘only after the intervention’					
Variable	No (*N* = 483)	Yes (*N* = 100)	OR	95% CI	*P*
**Age (years)**	39.5 ± 6.9	40.7 ± 8.2	1.02	0.99–1.06	0.134
**Gender**	180 (37.3)	41 (41.0)			0.484
Men	303 (62.7)	59 (59.0)	1.17	0.75–1.82	
Women			1.00	Reference	
**Place of residence**	158 (32.7)	45 (45.0)			0.020
Rural areas	325 (67.3)	55 (55.0)	1.68	1.09–2.61	
Urban areas			1.00	Reference	
**Ethnicity**	36 (7.5)	14 (14.0)			0.036
Roma/Egyptian	447 (92.5)	86 (86.0)	2.02	1.05–3.91	
Ethnic Albanian			1.00	Reference	

a Odds ratios (ORs: positive changes vs. no positive changes), their 95% confidence intervals (95% CIs) and *P*-values from crude/unadjusted binary logistic regression.

b Mean values ± standard deviations.

c Number and their respective ‘column’ percentages (in parentheses).

Provision of home-made foods to children for eating at school ‘only after the intervention’ was evidenced in 100 (17%) of participants (table 1, lower panel). There were no significant differences regarding age or gender, but a positive and significant association with rural residence (OR = 1.7, 95% CI = 1.1–2.6) and especially with Roma/Egyptian minority (OR = 2.0, 95% CI = 1.1–3.9).

## Discussion

We found a significant improvement in healthy nutritional practices among Albanian parents/caregivers benefiting from community-based health promotion campaigns coupled with multi-component online interventions. After the intervention, overall, there was an increase of more than 11% in the prevalence of home cooking and/or provision of home-made foods to children for eating at school. Engagement in healthy nutritional practices ‘only after the intervention’ was higher among parents/caregivers pertinent to Roma/Egyptian communities compared to their Albanian counterparts.

Our findings regarding the effectiveness of health promotion programs for inducement of positive changes among parents/caregivers are compatible with several previous reports which have convincingly shown that nutritional knowledge of family/caregivers, along with other interventions, can lead to healthy food choices for their children.^8^^,^^9^ Hence, improving family nutritional knowledge and creating a supportive family environment for children should be essential components in all interventions aiming at improving nutritional practices for children of low- and middle-income countries,^8^^,^^9^ including Albania.

For industrialized countries, it has been argued that children who bring food from home have poorer nutrition compared to their counterparts who consume the foods provided in school meal programs.^10^ However, for countries like Albania, bringing food from home is crucial for children, as neither free nor paid meals are offered in school settings. Hence, for Albanian children, consumption of homemade foods is by far healthier than the ultra-processed food easily accessible in the vicinities of school premises.^6^

Notably, our finding regarding male predominance in home cooking only after the intervention should be considered in upcoming health promotion programmes in Albania and beyond.

Conversely, our findings related to a higher improvement of nutritional practices ‘only after the intervention’ among parents/caregivers pertinent to Roma/Egyptian communities are mainly related to a lower baseline (i.e. before the intervention) prevalence of healthy behaviours in these categories compared with their ethnic Albanians counterparts.

Limitations of this study may involve sample representativeness, the possibility of reporting bias, and the study design.

Nonetheless, our findings from Albania indicate that multi-component interventions consisting of ‘onsite’ community-based health promotion campaigns coupled with ‘online’ interventions are effective means of promoting positive nutritional practices among parents/caregivers. In particular, in developing countries, several vulnerable/marginalized population categories may benefit the most, especially the minority groups including Roma/Egyptian communities.

Policymakers and decision-makers in Albania and elsewhere should expand the implementation of health promotion campaigns which are seemingly effective for increasing the engagement of parents/caregivers in healthy nutritional practices.

## Data Availability

The data presented in this study are available on request from the corresponding author.
